# Missing pieces, new patterns: the impact of association football international call-ups on team offensive and defensive performance indicators

**DOI:** 10.3389/fpsyg.2025.1697146

**Published:** 2026-01-22

**Authors:** João Campos, Bruno Gonçalves, Bruno Travassos, Nuno Mateus, Rafael Ballester Lengua, Bruno Figueira, Sigrid Olthof, Diogo Coutinho

**Affiliations:** 1 Department of Physical Education and Sports Sciences, University of Maia (UMAIA), Maia, Portugal; 2 Universidade de Évora, Escola de Saúde e Desenvolvimento Humano, Departamento de Desporto e Saúde, Évora, Portugal; 3 Universidade de Évora, Comprehensive Health Research Centre (CHRC), Évora, Portugal; 4 FPF Academy, Portuguese Football Federation, Oeiras, Portugal; 5 Research Center in Sports Sciences, Health Sciences and Human Development, CIDESD, Vila Real, Portugal; 6 Department of Sports Sciences, University of Beira Interior, Covilhã, Portugal; 7 Department of Sports Sciences, Exercise and Health, University of Trás-os-Montes and Alto Douro, Vila Real, Portugal; 8 Faculty of Physical Education and Sport Sciences, Catholic University of Valencia “San Vicente Mártir”, Torrent, Valencia, Spain; 9 Research Institute for Sport and Exercise and Sciences, Liverpool John Moores University, Liverpool, United Kingdom

**Keywords:** defensive performance indicators, International call-ups, offensive performance indicators, players absence, scouting, tournaments

## Abstract

**Introduction:**

To examine how mid-season international call-ups (AFCON and AFC Asian Cup) affect club performance across offensive, defensive, and playing-style key performance indicators (KPIs).

**Methods:**

A non-participant observational study analyzed 522 league matches from 58 teams in Europe’s top five leagues (2023–2024). For teams losing players to international duty (*n* = 130 players across positions), club matches were grouped into three phases: PRE (three matches before), INT-CUP (three during absences), and POST (three after return). Wyscout-derived KPIs covered ball possession, goal scoring, offensive play, set pieces, and defensive actions. Non-parametric repeated-measures ANOVA (*p* < .05) and Cohen’s d quantified differences.

**Results:**

INT-CUP showed clear improvements in ball-possession KPIs versus PRE and POST: higher total, successful, frontal, lateral, and backward passes; more progressive and deep completed passes; more crosses; and greater passes per possession, alongside shorter average passing length (all *p* ≤ .05; small–moderate effects). Goal-scoring output increased during INT-CUP (more shots, shots on target—including from outside the box—and goals vs PRE; more goals vs POST; *p* ≤ .05). Offensive penetration also rose (penalty-area entries and area touches; *p* ≤ .05), and positional attacks ending in shots were more frequent during INT-CUP (*p* = .015). Set-piece KPIs did not differ meaningfully. Defensively, PRE exceeded POST in duels, duels won, and defensive duels (*p* ≤ .05), while conceded goals were broadly unchanged across phases.

**Discussion:**

Contrary to expectations, international absences coincided with a more possession-oriented style and enhanced attacking output, without compromising defensive outcomes. Effects between PRE and POST were modest, suggesting tactical adaptations during absences can sustain or even improve offensive efficiency. Coaches may leverage forced rotations to explore possession-based structures that preserve defensive stability.

## Introduction

Association football (soccer) is the world’s most popular and widely influential sport, played and followed around the globe (Hughson, 2010). As success in soccer increasingly relies on securing competitive advantages, the growing prominence of data-driven analyses becomes essential for improving decision-making and performance processes (Olthof and Davis, 2025). In this context, match analysis using performance statistics has become indispensable for coaches and analysts aiming to improve team outcomes (Stafylidis et al., 2024). Modern technological advancements now enable the collection of massive amounts of match data, ranging from player tracking to detailed event logs, on a match-by-match basis (Goes-Smit et al., 2020). By objectively quantifying on-field actions, teams can identify strengths and weaknesses more precisely and implement targeted interventions to enhance performance (Herold et al., 2022).

One of the core concepts in match analysis is the use of key performance indicators (KPIs), which are statistical metrics that capture critical technical-tactical aspects of play (Herold et al., 2021; Phatak et al., 2022; Plakias et al., 2024). KPIs are selected variables that capture key performance facets, including tactical situations and playing styles, which are understood to influence success in soccer (Herold et al., 2021; Hughes and Franks, 2010; Hughes and Lovell, 2019). With the advent of sophisticated data providers (e.g., Wyscout, Opta), teams and researchers now have access to dozens of KPIs describing every pass, shot, duel, and more across each game (Otero-Saborido et al., 2021; Pappalardo et al., 2019b). These metrics allow for systematic comparisons of players and teams, providing a common language to evaluate performance (Franks et al., 2016). For example, shots on target and ball possession-related variables (i.e., total passes, accuracy of passes, long passes) are considered key factors affecting match outcomes and distinguishing high-performing teams (Rocha-Lima et al., 2021). The reliability and depth of such data systems have improved substantially in recent years, making it feasible to profile team playing styles and effectiveness with a high degree of confidence (Goes-Smit et al., 2020). Consequently, performance analysis in soccer has evolved from simple box-score statistics to complex, multidimensional data evaluations that inform both scouting and in-game strategy (Stafylidis et al., 2024).

Prior research has repeatedly shown that certain technical-tactical indicators correlate strongly with team success (Oliva-Lozano et al., 2023). In particular, offensive performance metrics have been highlighted as key discriminators between winning and losing teams. Successful teams tend to produce more shots (especially shots on target) and convert them efficiently into goals than unsuccessful teams (Castellano et al., 2012; Dufour et al., 2017; Kubayi and Larkin, 2022). For instance, in international tournaments, top-performing national teams executed a higher number of shots on goal and achieved superior goal conversion rates compared to less successful teams (Delgado-Bordonau et al., 2013; Fan et al., 2023). Analyses of elite club competitions align with these patterns, showing that winning teams typically outperform their opponents in goal-scoring indicators, such as total goals and shot accuracy (Delgado-Bordonau et al., 2013; Stafylidis et al., 2024). Notably, it is not just the quantity of shots but also their quality and efficiency that matter; scoring efficiency (goals per shot ratio) has been shown to be a crucial factor in differentiating match outcomes (Stafylidis et al., 2024). For example, in the 2010 World Cup, the most successful squads not only created more scoring opportunities but also conceded significantly fewer goals than eliminated teams, underscoring the importance of capitalizing on chances while minimizing opponents’ chances (Evangelos et al., 2018). Collectively, these studies indicate that successful outcomes in soccer are largely driven by superior attacking performance and efficient goal-scoring.

Effective passing and possession-based play represent important KPIs that are strongly linked to successful team performance (Plakias et al., 2024; Rein et al., 2017). Passing sequences are the foundation of a team’s ability to control the game, and research indicates that successful teams display higher passing volume and accuracy during matches (Plakias et al., 2024). Successful passing has been identified as a key component of soccer performance in a dual sense: it prevents the opponent from using the ball (limiting the opposition’s chances to score) and provides a platform for one’s own team to build attacks leading to shots (Herold et al., 2021; Rein et al., 2017). In the English Premier League and other top competitions, winning teams distinguish themselves by completing more passes (especially in the opposition half) and stringing together longer passing sequences, reflecting an ability to dominate possession and territory (Rocha-Lima et al., 2021). Furthermore, a high ball possession percentage—particularly in the attacking third—and a greater number of progressive or penetrating passes have been associated with more frequent scoring opportunities (Stafylidis et al., 2024). In essence, teams that manage to retain the ball and advance it cohesively up the field tend to create better conditions for scoring while denying the opposition the chance to impose their game.

While offensive prowess has understandably been a focal point, defensive performance indicators are also vital to success (Türegün, 2019), although they have received comparatively less attention in the literature. Football is a dynamic sport where the transition from attack to defense (and vice versa) can be decisive. Studies have noted that a large proportion of goals, high-risk scoring chances, and creating more opportunities occur immediately after possession turnovers, during the phase of defensive transition. For this reason, metrics such as successful defensive duels, interceptions, and recoveries (regaining possession from the opponent) can be crucial in preventing goals (Casal et al., 2020; Fernandes et al., 2020). Some analyses of elite tournaments suggest that less successful teams perform worse in defensive aspects; for example, they may win fewer duels or concede more shots (Castellano et al., 2012). However, consistently quantifying defensive effectiveness is challenging due to the lack of defensive metrics for soccer players (Hvattum, 2020). This gap has been attributed to the complexity and context-dependence of defensive actions (e.g., a team leading a match might deliberately concede possession and thus record more defensive actions without jeopardizing the result) (Phatak et al., 2022). Nonetheless, there is growing recognition that balanced success in football requires excellence in both scoring and preventing goals, warranting the inclusion of defensive KPIs in any comprehensive performance analysis.

Despite these valuable insights into performance indicators, there remains a notable gap in the literature regarding situational factors that disrupt team performance, particularly the temporary absence of key players (Perez, 2021). Modern elite football is characterized by congested competition calendars, where club fixtures often overlap with international tournaments (Julian et al., 2021). A pertinent example is the mid-season scheduling of major continental competitions, such as the Africa Cup of Nations (AFCON) and the AFC Asian Cup (AC), which traditionally take place in January–February (Acha-anyi, 2023; Ge et al., 2022). With the global migration of football talent, many top European clubs have players who are called up to represent their national teams in these tournaments. Recent statistics showed that over 270 African players were contracted to clubs in Europe’s “Big Five” leagues (England, Spain, Germany, Italy, and France) during the 2023–2024 season (Perez, 2021). When such players depart for several weeks to play for their countries, their clubs are forced to compete without them in league matches during that period (Perez, 2021). This raises an important question for both practitioners and researchers: How does the absence of key players on international duty impact a team’s performance on the pitch?

Intuitively, one might expect that losing important players would hinder a team’s performance, and there is some evidence to support this (Perez, 2021). For instance, a recent analysis focusing on AFCON absences found a *relatively* small but noticeable decline in European club performance (e.g., points gained in league matches) when players were away at the tournament (Perez, 2021). However, that study noted that the effect was not uniform across all leagues and tended to diminish after accounting for the individual abilities of the absent players (Perez, 2021). In other words, teams with deeper squads and greater resources may cope better with such absences than less well-resourced teams (Perez, 2021). Beyond match outcomes, there is very little empirical research evaluating how player absences influence the technical and tactical performance metrics of teams (Perez, 2021). Most of the literature on team performance disruptions has focused on injuries or fatigue resulting from congested schedules (Julian et al., 2021), rather than on performance indicator trends when players temporarily leave for external competitions. Therefore, the present study aims to assess the impact of mid-season international tournament absences on team performance, using a comprehensive set of technical-tactical KPIs. In particular, the study focuses on clubs from the top five European leagues during the 2023–2024 season that lost players to the AFCON (and, concurrently, the AC) and compares the teams’ performance in league matches before, during, and after these absences.

## Methods

### Match sample and data collection

The present study used a non-participant observational design and analyzed 522 games from 58 professional teams competing in the first divisions of England, Spain, Germany, Italy, and France during the 2023—2024 season. Data were collected from 130 players across different playing positions (see Table 1.). All competitive matches included in this study involved teams with players called up to represent their national teams in the AFCON and AC. Players who played an average of less than 90 min in matches preceding the AFCON and AC (INT-CUP); players who were injured or ill before, during, or after the INT-CUP; players who were transferred; and teams that played fewer than three matches during the absence of players called up for the INT-CUP were excluded from this study. All data were gathered from the online platform Wyscout (Wyscout Spa, Chiavari, Italy), which is a reliable data-based system (Pappalardo et al., 2019a). All data were extracted via Wyscout’s match-event API using standardized filters, and 10% of the matches were double-checked for accuracy. As the data were automatically captured by the Wyscout system and not manually coded by the researchers, traditional intra- and inter-rater reliability coefficients (e.g., Cohen’s kappa, ICC) are not applicable to the present study. Previous independent validations of Wyscout have reported high levels of accuracy and consistency in event detection and classification (González Rodenas et al., 2019a,b), supporting the reliability of the data source.

**Table 1:** Number of players who left to represent their national teams, categorized by position.

Position	Number of players
Goalkeeper	2
Centre back	25
Fullback	16
Defensive midfielder	14
Central midfielder	16
Offensive midfielder	6
Winger	37
Forward	14

As all data were open-access and anonymized, formal ethical approval was not required.

### Procedures

Data were collected and organized in a spreadsheet using Microsoft Excel, covering nine matches for each team with players called up for national duties. These matches were divided into three phases: (i) PRE, consisting of three matches during which the players were available for their club; (ii) INT-CUP, corresponding to three matches during their absence; and (iii) POST, consisting of three matches following their return to the club. Comprehensive information related to each match was also recorded, including the country, competition, division, team, team quality, opponent quality, the maximum number of players leaving for international duties, the number of player absences, and the specific phase during which the absences occurred.

Variables were selected from the Wyscout database and organized according to different categories: (i) ball possession, (ii) goal scoring, (iii) offensive play, (iv) playing style, (v) set pieces, and (vi) defensive performance. The categories and operational definitions are presented in Table 2 (Hudl, Agile Sports Technologies, 2024; Liu et al., 2015; Lago-Peñas et al., 2010; The International Football Association Board (IFAB), 2024; Yi et al., 2019a).

**Table 2:** List of dependent variables considered (definitions based on the Wyscout glossary).

Groups	Events (unit)	Operational definition
Ball possession	Total passes (n)	The aggregate number of passes attempted during a match
	Successful passes (n)	The number of passes that successfully reach the intended recipient without interception.
	Frontal passes (n)	Total number of passes at a 90° angle rotated by 45° facing forwards.
	Successful frontal passes (n)	Total number of accurate forward passes.
	Lateral passes (n)	Total number of passes at two 90° angles, rotated by 45° facing sideways, longer than 12 m.
	Successful lateral passes (n)	Total number of accurate lateral passes. Also available as a percentage.
	Backward passes (n)	Total number of passes at a 90° angle rotated by 45° facing backwards.
	Successful backward passes (n)	Total number of accurate backward passes.
	Long passes (n)	A ground pass longer than 45 m or a high pass longer than 25 m.
	Successful long passes (n)	A long pass is deemed successful when a teammate performs the next touch.
	Deep completed passes (n)	A Cross that is targeted to the zone within 20 m of the opponent’s goal.
	Final third passes (n)	Any pass played from outside the final third whose next touch occurs within the final third.
	Final third successful passes (n)	A pass into the final third is considered successful when a teammate makes the next touch.
	Progressive passes (n)	A forward pass is intended to move the team substantially closer to the opponent’s goal.
	Successful progressive passes (n)	A progressive pass is considered successful when a teammate makes the next touch.
	Average passes per possession (n)	Average number of passes in an open-play possession.
	Average passing length (m)	Average length of passes.
	Crosses (n)	Any ball sent into the opposition team’s area from a wide position.
	Successful crosses (n)	A cross is considered successful if the next touch is by a teammate.
Goal scoring	Total shots (n)	Number of all shots attempted in the timeframe.
	Shots on target (n)	An attempt on goal that either required intervention to prevent it from entering the net or was on target and would have scored without diversion.
	Shots on target: outside the penalty area (n)	The total number of on-target shots taken from outside the opponent’s penalty area.
	Average shooting distance (m)	The average distance from the team’s own goal to the opponent’s goal for all shots.
	Goals (n)	A goal is scored when the entire ball crosses the goal line between the posts and under the crossbar, without any infringement by the scoring team.
	Goals conceded (n)	Total number of goals conceded.
	Shots against (n)	A shot on target faced by the goalkeeper
	Shots against on target (n)	Total number of shots that were on target.
Offensive play	Penalty area entries (n)	Total number of penalty area entries (via pass, cross, or carry).
	Area touches (n)	An action (a pass or a touch) that happens in the opponent’s penalty area.
	Offensive duels (n)	A ground duel for the player in possession of the ball.
	Successful offensive duels (n)	A duel is considered successful if it is followed by the same attacking player advancing the ball, an attacking teammate moving the ball closer to the opponent’s goal, or a defensive foul.
	Ball losses (n)	Any action that ends a possession of the current team.
Playing style	Positional attacks (n)	An open-play attack that is not a counter-attack.
	Positional Attacks with shots (n)	Total number of positional attacks that included a shot.
	Counter-attacks (n)	A possession turnover in which the team rapidly transitions from defense to attack to exploit the opponent’s disorganized defensive shape.
	Counter-attacks with shots (n)	Total number of counter-attacks that included a shot.
Set pieces	Set pieces (n)	Events where play resumes after a stoppage, such as a foul or the ball going out.
	Set pieces ending in shots (n)	Total number of set piece attacks that included a shot during the possession.
	Corners (n)	Ball goes out of play for a corner kick.
	Corners ending in shots (n)	A team shot occurring within 14 s of a corner awarded to the same team.
	Free kick (n)	Free kicks, direct or indirect, awarded to the opposing team for an offence by a player, substitute, substituted or sent-off player, or team official.
	Free kick ending in shots (n)	A shot taken from a direct free kick or immediately following an indirect free kick.
	Goal kicks (n)	A goal kick is awarded when the ball, last touched by an attacking player, crosses the goal line without resulting in a goal.
Defensive performance	Ball recoveries (n)	Any action that ends the opponent’s possession and initiates possession for the team.
	Duels (n)	A contest between two players to gain, advance, or redirect the ball.
	Duels won (n)	Total number of duels won.
	Defensive duels (n)	An attempt by a player to dispossess an opponent and halt the attack.
	Defensive duels won (n)	A defensive duel is won when the defender halts the attacker’s progress without committing a foul.
	Aerial duels (n)	Two or more players from opposing teams jump to compete for the ball.
	Successful aerial duels (n)	An aerial duel is won by the first player to touch the ball or by the player who is fouled.
	Interceptions	An action in which a player intercepts the ball by anticipating an opponent’s shot, pass, or cross.
	Clearances (n)	An action, typically a pass, where a player clears the ball—forward without a target or for a throw-in/corner—choosing safety over control.
	Fouls (n)	Any infringement that is penalized as foul play by a referee.
	Yellow cards (n)	Yellow cards issued to a player for fouls, persistent infringements, handball, dangerous play, or similar offenses.
	Red cards (n)	Disciplinary action by the referee that is indicated by showing a red card.
	PPDA (n)	A metric to quantify high pressing intensity in the final 60% of the field.

n, number; PPDA, Passes Per Defensive Action.

Offensive performance variables, including progressive passes, deep completed passes, final third entries, and penalty-area touches, were selected because they represent actions that directly contribute to advancing the ball, breaking defensive lines, and creating scoring opportunities, which are widely considered essential components of attacking effectiveness (Guimarães et al., 2021; Prieto González et al., 2024). Defensive performance variables, such as duels, defensive duels won, interceptions, and passes per defensive action (PPDA), were chosen to capture a team’s ability to disrupt the opponent’s build-up play, apply pressure, and recover possession. PPDA provides insight into pressing intensity, while metrics such as progressive passes quantify forward progression and territorial gain. Together, these indicators create a comprehensive framework for evaluating how player absences influence both the creation and prevention of goal-scoring opportunities (Bekkers, 2024; Clemente et al., 2016).

### Statistical analysis

All variables were inspected for outliers and tested for normality using visual inspection and the Shapiro–Wilk test. As several variables violated the assumption of normality and the study followed a repeated measures design (same teams observed in PRE, INT-CUP, and POST), differences in KPIs across the phases were analyzed using non-parametric repeated measures ANOVA (Friedman test). When a significant main effect was detected, pairwise *post hoc* comparisons (PRE vs. INT-CUP, PRE vs. POST, and INT-CUP vs. POST) were performed using the Durbin–Conover test. In addition, Cohen’s d effect sizes were calculated as complementary information. All statistical analyses were performed using the Jamovi Project software (Computer Software Version 2.3.21.0, 2023), with a *p*-value of < 0.05 indicating statistical significance. Complementarily, pairwise differences were assessed by examining differences in group means, expressed in raw data units with 95% confidence intervals (CI). Thresholds for effect size statistics were as follows: <0.2, trivial; <0.6, small; <1.20, moderate; <2.0, large; and >2.0, very large (Hopkins et al., 2009).

## Results

### Offensive performance indicators

**Figure 1:**
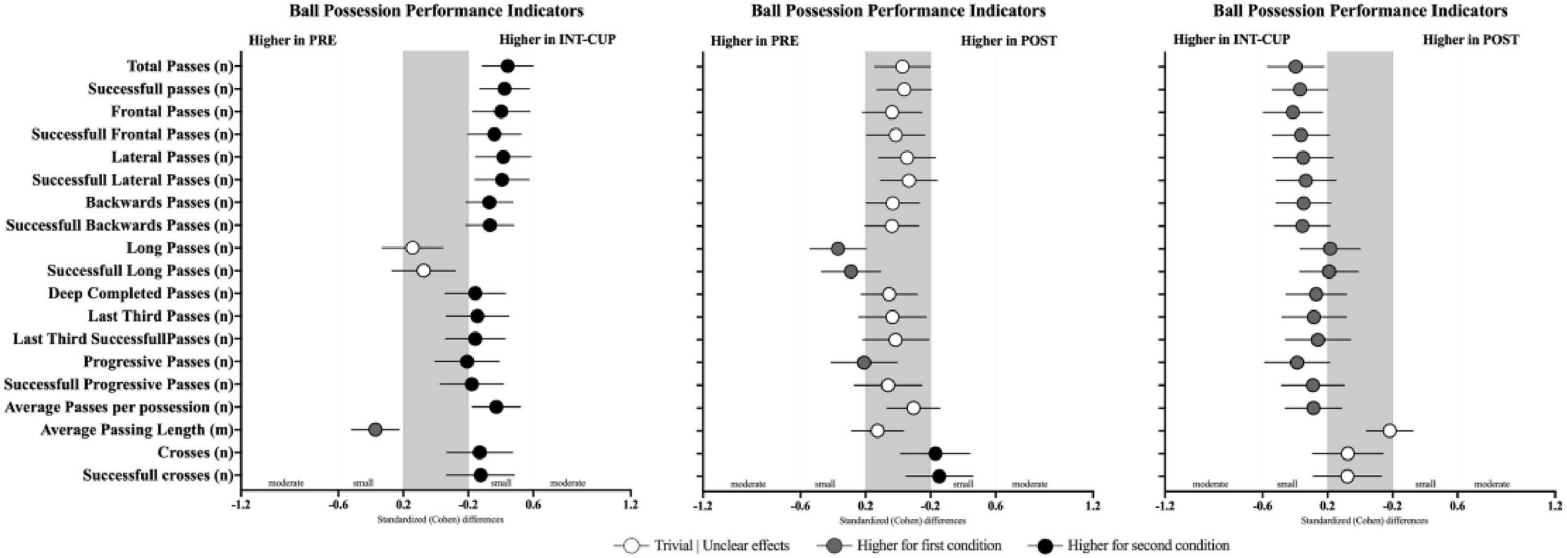
Standardized (Cohen’s *d*) differences in passing-related variables across the conditions (PRE vs. INT-CUP, PRE vs. POST, and INT-CUP vs. POST). Error bars indicate uncertainty in the true mean changes with 95% confidence intervals.

**Figure 2:**
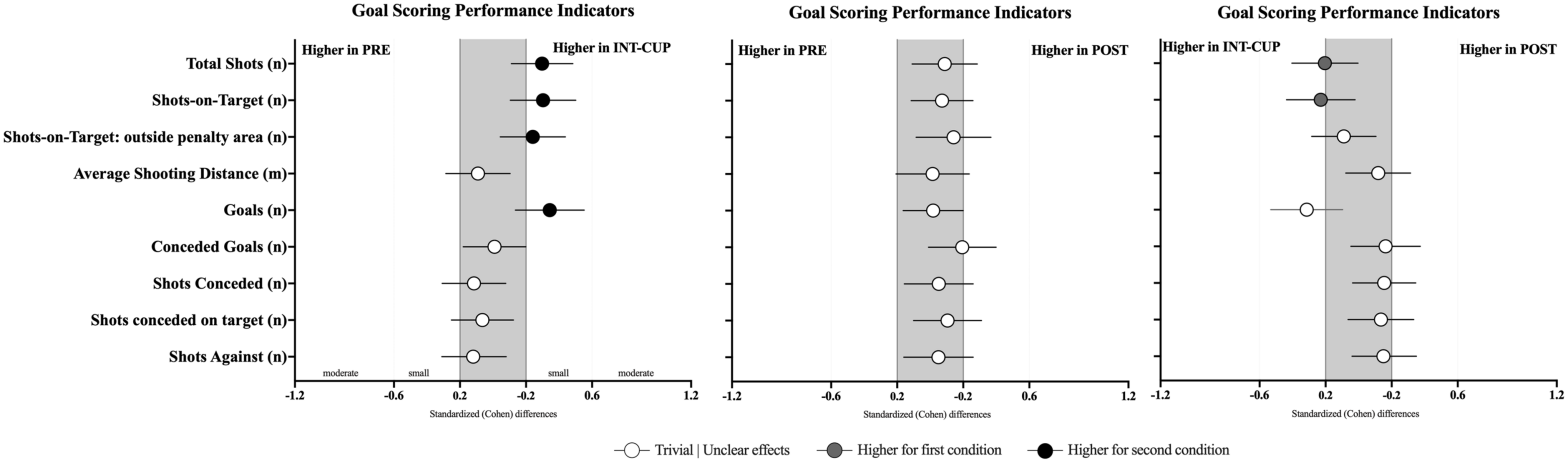
Standardized (Cohen’s *d*) differences in goal-scoring variables across the conditions (PRE vs. INT-CUP, PRE vs. POST, and INT-CUP vs. POST). Error bars indicate uncertainty in the true mean changes with 95% confidence intervals.

**Figure 3:**
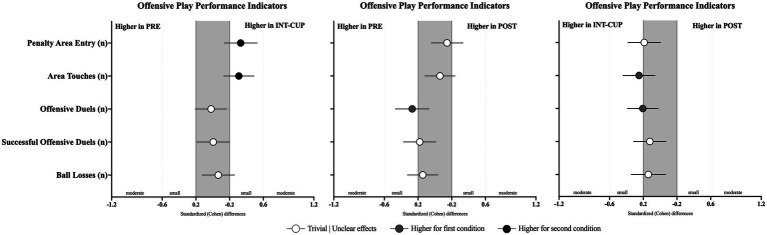
Standardized (Cohen’s *d*) differences in other offensive play variables across the conditions (PRE vs. INT-CUP, PRE vs. POST, and INT-CUP vs. POST). Error bars indicate uncertainty in the true mean changes with 95% confidence intervals.

Differences in teams’ offensive performance between the conditions (PRE vs. INT-CUP; PRE vs. POST; and INT-CUP vs. POST) are presented in Table 3, Figures 1–3. The ball possession-related variables were the performance indicators that showed the largest differences between the conditions. Accordingly, statistically significant differences between teams were found for total passes (*n*, *X*^2^ = 17.2, *p* = 0.008), successful passes (*n*, *X*^2^ = 16.6, *p* = 0.008) frontal passes (*n*, *X*^2^ = 20.1, *p* = 0.008), successful frontal passes (*n*, *X*^2^ = 11.7, *p* = 0.008), lateral passes (*n*, *X*^2^ = 14.6, *p* = 0.008), successful lateral passes (*n*, *X*^2^ = 11.7, *p* = 0.008), backward passes (*n*, *X*^2^ = 14.6, *p* = 0.008), successful backward passes (*n*, *X*^2^ = 10.2, *p* = 0.008), long passes (*n*, *X*^2^ = 8.08, *p* = 0.008), successful long passes (*n*, *X*^2^ = 6.22, *p* = 0.008), deep completed passes (*n*, *X*^2^ = 6.98, *p* = 0.008), progressive passes (*n*, *X*^2^ = 7.99, *p* = 0.008), average passes per possession (*n*, *X*^2^ = 15.6, *p* = 0.008), average passing length (m, X^2^ = 20.3, *p* = 0.008), crosses (*n*, *X*^2^ = 8.88, *p* = 0.008), and successful crosses (*n*, *X*^2^ = 8.00, *p* = 0.008). In this respect, the INT-CUP phase revealed higher values than the PRE phase for total passes (*p* = 0.001; ES = 0.44 [0.29; 0.6]), successful passes (*p* = 0.001; ES = 0.42 [0.27; 0.58]), frontal passes (*p* = 0.002; ES = 0.4 [0.23; 0.58]), successful frontal passes (*p* = 0.015; ES = 0.36 [0.2; 0.52]), lateral passes (*p* < 0.001; ES = 0.41 [0.24; 0.58]), successful lateral passes (*p* = 0.002; ES = 0.41 [0.24; 0.57]), backward passes (*p* = 0.001; ES = 0.33 [0.18; 0.48]), successful backward passes (*p* < 0.001; ES = 0.33 [0.19; 0.48]), deep completed passes (*p* = 0.031; ES = 0.24 [0.06; 0.43]), progressive passes (*p* = 0.005; ES = 0.19 [0; 0.39]), average passes per possession (*p* < 0.001; ES = 0.37 [0.23; 0.52]), crosses (*p* = 0.003; ES = 0.27 [0.07; 0.47]), and successful crosses (*p* = 0.013; ES = 0.28 [0.07; 0.48]). In contrast, average passing length was lower (*p* < 0.001; ES = −0.37 [−0.52; −0.23]). The INT-CUP condition also showed higher values than the POST condition for total passes (*p* < 0.001; ES = −0.4 [−0.57; −0.22]), successful passes (*p* < 0.001; ES = −0.37 [−0.54; −0.2]), frontal passes (*p* < 0.001; ES = −0.41 [−0.6; −0.23]), successful frontal passes (*p* < 0.001; ES = −0.36 [−0.54; −0.19]), lateral passes (*p* = 0.002; ES = −0.35 [−0.53; −0.16]), successful lateral passes (*p* = 0.017; ES = −0.33 [−0.52; −0.15]), backward passes (*p* = <001; ES = −0.35 [−0.52; −0.18]), successful backward passes (*p* < 0.001; ES = −0.35 [−0.53; −0.18]), long passes (*p* = 0.036; ES = −0.18 [−0.37; 0.00]), deep completed passes (*p* = 0.016; −0.27 [−0.46; −0.08]), average passes per possession (*p* = 0.007; −0.29 [−0.46; −0.11]), and average passing length (*p* = 0.008; 0.18 [0.04; 0.32]). Regarding the comparison between PRE and POST, the PRE phase revealed higher mean values for long passes (*p* = 0.007; ES = −0.37 [−0.54; −0.2]), successful long passes (*p* = 0.014; ES = −0.29 [−0.47; −0.11]), average passing length (*p* = 0.054; ES = −0.13 [−0.29; 0.03]), and successful crosses (*p* = 0.015; ES = 0.25 [0.05; 0.46]).

**Table 3:** Descriptive and inferential statistics of offensive performance indicators across the conditions (PRE, INT-CUP, and POST).

Variables	PRE	INT-CUP	POST	Difference in means (raw; ±95% CL)			*P*	ES with 95% CI		
	(M ± SD)	(M ± SD)	(M ± SD)	PRE vs. INT-CUP	PRE vs. POST	INT-CUP vs. POST		PRE	INT-CUP	POST
Ball possession variables										
Total passes (n)	457.03 ± 124.20	518.53 ± 145.65	463.62 ± 143.3	61.50; ±21.72	3.69 ± 23.63	−54.91; ±24.07	**<0.001**	0.44 [0.29; 0.60]	0.03 [−0.14; 0.20]	−0.40 [−0.57; −0.22]
Successful passes (n)	386.53 ± 121.61	443.86 ± 142.46	393.74 ± 139.21	57.33; ±20.60	5.12 ± 22.71	−50.12; ±23.14	**<0.001**	0.42 [0.27; 0.58]	0.04 [−0.13; 0.21]	−0.37 [−0.54; −0.20]
Frontal passes (n)	149.67 ± 29.03	162.69 ± 34.46	149.33 ± 32.77	13.02; ±5.66	−1.18; ±5.92	−13.36; ±5.92	**<0.001**	0.40 [0.23; 0.58]	−0.04 [−0.22; 0.15]	−0.41 [−0.60; −0.23]
Successful frontal passes (n)	112.42 ± 28.85	123.75 ± 33.23	112.32 ± 31.64	11.33; ±5.17	−0.44; ±5.64	−11.43; ±5.55	**0.003**	0.36 [0.2; 0.52]	−0.01 [−0.19; 0.17]	−0.36 [−0.54; −0.19]
Lateral passes (n)	164.70 ± 60.37	191.94 ± 69.24	168.97 ± 66.04	27.24; ±11.18	3.69; ±11.48	−22.97; ±12.18	**<0.001**	0.41 [0.24; 0.58]	0.06 [−0.12; 0.23]	−0.35 [−0.53; −0.16]
Successful lateral passes (n)	147.80 ± 58.50	173.72 ± 67.19	152.55 ± 63.81	25.91; ±10.63	4.28; ±10.99	−21.17; ±11.63	**0.006**	0.41 [0.24; 0.57]	0.07 [−0.11; 0.24]	−0.33 [−0.52; −0.15]
Backward passes (n)	71.24 ± 23.04	79.52 ± 26.43	70.81 ± 25.31	8.28; ±3.66	−0.82; ±4.13	−8.71; ±4.25	**<0.001**	0.33 [0.18; 0.48]	−0.03 [−0.20; 0.13]	−0.35 [−0.52; −0.18]
Successful backward passes (n)	67.24 ± 22.29	75.40 ± 25.93	66.70 ± 24.74	8.16; ±3.61	−0.94; ±4.02	−8.70; ±4.19	**<0.001**	0.33 [0.19; 0.48]	−0.04 [−0.20; 0.13]	−0.35 [−0.53; −0.18]
Long passes (n)	45.46 ± 11.11	43.83 ± 12.11	41.75 ± 10.51	−1.63; ±2.11	−4.18; ±1.95	−2.09; ±2.09	**0.018**	−0.14 [−0.33; 0.04]	−0.37 [−0.54; −0.20]	−0.18 [−0.37; 0.00]
Successful long passes (n)	25.60 ± 7.61	25.05 ± 7.51	23.64 ± 6.88	−0.56; ±1.43	−2.13; ±1.35	−1.41; ±1.32	**0.045**	−0.08 [−0.27; 0.12]	−0.29 [−0.47; −0.11]	−0.19 [−0.37; −0.01]
Deep completed passes (n)	8.10 ± 5.43	9.45 ± 6.25	7.95 ± 4.83	1.35; ±1.03	−0.31; ±0.97	−1.50; ±1.03	**0.031**	0.24 [0.06; 0.43]	−0.06 [−0.23; 0.12]	−0.27 [−0.46; −0.08]
Final third passes (n)	52.02 ± 17.27	56.68 ± 19.49	51.53 ± 17.29	4.66; ±3.49	−0.63; ±3.73	−5.15; ±3.58	0.169	0.26 [0.06; 0.45]	−0.03 [−0.24; 0.17]	−0.28 [−0.48; −0.09]
Final third successful passes (n)	37.02 ± 16.04	41.18 ± 18.60	36.75 ± 16.27	4.16; ±3.14	−0.25; ±3.47	−4.43; ±3.42	0.084	0.24 [0.06; 0.43]	−0.01 [−0.22; 0.19]	−0.26 [−0.46; −0.06]
Progressive passes (n)	70.86 ± 14.44	73.63 ± 15.18	68.06 ± 13.15	2.77; ±2.82	−3.01; ±2.91	−5.56; ±2.87	**0.018**	0.19 [0.00; 0.39]	−0.21 [−0.41; −0.01]	−0.39 [−0.59; −0.19]
Successful progressive passes (n)	50.25 ± 15.11	53.57 ± 15.72	49.22 ± 13.77	3.32; ±2.91	−0.93; ±3.11	−4.34; ±2.90	0.071	0.22 [0.03; 0.42]	−0.06 [−0.27; 0.15]	−0.29 [−0.48; −0.10]
Average passes per possession (n)	4.49 ± 1.31	5.03 ± 1.50	4.62 ± 1.45	0.53; ±0.21	0.14; ±0.23	−0.41; ±0.25	**<0.001**	0.37 [0.23; 0.52]	0.09 [−0.07; 0.26]	−0.29 [−0.46; −0.11]
Average passing length (m)	19.20 ± 1.62	18.61 ± 1.50	18.90 ± 1.57	−0.58; ±0.23	−0.20; ±0.25	0.29; ±0.22	**<0.001**	−0.37 [−0.52; −0.23]	−0.13 [−0.29; 0.03]	0.18 [0.04; 0.32]
Crosses (n)	14.25 ± 6.48	16.13 ± 6.99	15.61 ± 7.27	1.88; ±1.41	1.60; ±1.48	−0.52; ±1.50	**0.012**	0.27 [0.07; 0.47]	0.23 [0.02; 0.44]	−0.08 [−0.29; 0.14]
Successful crosses (n)	4.53 ± 2.92	5.39 ± 3.31	5.15 ± 3.03	0.86; ±0.65	0.79; ±0.64	−0.24; ±0.65	**0.018**	0.28 [0.07; 0.48]	0.25 [0.05; 0.46]	−0.08 [−0.29; 0.13]
Goal-scoring variables										
Total shots (n)	11.93 ± 5.20	13.47 ± 5.49	12.41 ± 4.74	1.54; ±0.96	0.45; ±1.02	−1.06; ±1.04	**0.008**	0.30 [0.11; 0.48]	0.09 [−0.11; 0.28]	−0.20 [−0.40; 0.00]
Shots on target (n)	4.30 ± 2.46	5.11 ± 2.96	4.50 ± 2.47	0.80; ±0.53	0.19; ±0.50	−0.61; ±0.55	**0.025**	0.30 [0.11; 0.50]	0.07 [−0.12; 0.26]	−0.23 [−0.44; −0.02]
Shots on target: outside the penalty area (n)	1.11 ± 1.15	1.40 ± 1.25	1.29 ± 1.23	0.29; ±0.24	0.17; ±0.28	−0.11; ±0.24	**0.047**	0.24 [0.04; 0.44]	0.14 [−0.09; 0.37]	−0.09 [−0.28; 0.10]
Average shooting distance (m)	17.84 ± 2.84	17.59 ± 2.59	17.92 ± 2.99	−0.26; ±0.55	0.04; ±0.63	0.34; ±0.55	0.617	−0.09 [−0.29; 0.1]	0.01 [−0.21; 0.24]	0.12 [−0.08; 0.31]
Goals (n)	1.35 ± 1.17	1.82 ± 1.63	1.39 ± 1.24	0.47; ±0.29	0.02; ±0.25	−0.43; ±0.30	**0.003**	0.34 [0.14; 0.55]	0.02 [−0.16; 0.20]	−0.31 [−0.53; −0.10]
Goals conceded (n)	1.33 ± 1.14	1.34 ± 1.21	1.54 ± 1.31	0.01; ±0.23	0.24; ±0.25	0.20; ±0.26	0.62	0.01 [−0.18; 0.20]	0.19 [−0.01; 0.40]	0.16 [−0.05; 0.37]
Shots against (n)	11.78 ± 5.27	11.20 ± 4.85	11.98 ± 5.04	−0.59; ±0.98	0.26; ±1.06	0.79; ±0.97	0.261	−0.12 [−0.31; 0.08]	0.05 [−0.16; 0.26]	0.15 [−0.04; 0.35]
Shots against on target (n)	4.49 ± 2.43	4.33 ± 2.40	4.67 ± 2.67	−0.16; ±0.47	0.26; ±0.52	0.34; ±0.50	0.425	−0.06 [−0.25; 0.12]	0.10 [−0.10; 0.31]	0.13 [−0.06; 0.33]
Offensive play variables										
Penalty area entries (n)	23.51 ± 9.53	26.89 ± 11.07	24.97 ± 9.70	3.38; ±2.00	1.47; ±1.92	−1.92; ±1.97	**0.013**	0.33 [0.14; 0.53]	0.14 [−0.04; 0.33]	−0.19 [−0.38; 0.01]
Area touches (n)	19.06 ± 9.59	22.18 ± 11.11	19.68 ± 8.93	3.11; ±1.80	0.64; ±1.79	−2.50; ±1.92	**0.011**	0.31 [0.13; 0.49]	0.06 [−0.12; 0.24]	−0.25 [−0.44; −0.06]
Offensive duels (n)	72.17 ± 15.73	71.84 ± 16.74	68.49 ± 16.32	−0.32; ±3.08	−4.41; ±3.26	−3.36; ±3.01	0.058	−0.02 [−0.21; 0.17]	−0.27 [−0.47; −0.07]	−0.21 [−0.39; −0.02]
Successful offensive duels (n)	27.71 ± 7.74	27.75 ± 8.66	26.76 ± 7.95	0.05; ±1.59	−1.47; ±1.58	−0.99; ±1.57	0.17	0.01 [−0.19; 0.20]	−0.18 [−0.37; 0.01]	−0.12 [−0.31; 0.07]
Ball losses (n)	104.84 ± 16.39	106.01 ± 19.25	103.53 ± 16.94	1.17; ±3.39	−2.55; ±3.21	−2.48; ±3.60	0.446	0.07 [−0.13; 0.26]	−0.14 [−0.33; 0.04]	−0.14 [−0.34; 0.06]

Bold values indicate statistically significant differences between the conditions: (a) statistically significant differences between PRE and INT-CUP; (b) statistically significant differences between PRE and Post; (c) statistically significant differences between INT-CUP and POST.

Regarding the goal-scoring variables, statistically significant differences between the conditions were found for total shots (*n*, *X*^2^ = 9.64, *p* = 0.008), shots on target (*n*, *X*^2^ = 7.35, *p* = 0.025), shots on target from outside the penalty area (*n*, *X*^2^ = 6.12, *p* = 0.047), and goals (*n*, *X*^2^ = 11.7, *p* = 0.003). In general, higher offensive performance was observed in the INT-CUP condition, with a higher number of total shots (*p* = 0.002, ES with 95% confidence intervals: ES = 0.3 [0.11; 0.48]), shots on target (*p* = 0.007, ES = 0.3 [0.11; 0.50]), shots on target from outside the penalty area (*p* = 0.002, ES = 0.34 [0.14; 0.55]), and goals compared to the PRE condition. In addition, a higher number of goals was observed compared to the POST condition (*p* = 0.005, ES = −0.31 [−0.53; −0.10]).

Finally, regarding the offensive play variables, statistically significant differences between the conditions were observed for penalty area entries (*n*, *X*^2^ = 8.07, *p* = 0.013) and area touches (*n*, *X*^2^ = 8.93, *p* = 0.011). Accordingly, higher values of penalty area entries were found for INT-CUP compared to PRE (*p* = 0.005; ES = 0.33 [0.14; 0.53]) and POST (*p* = 0.003; ES = −0.19 [−0.38; 0.01]). In addition, a higher number of area touches (*p* = 0.003; ES = 0.31 [0.13; 0.49]) was observed in INT-CUP compared to PRE.

### Offensive playing style-related variables

**Figure 4:**
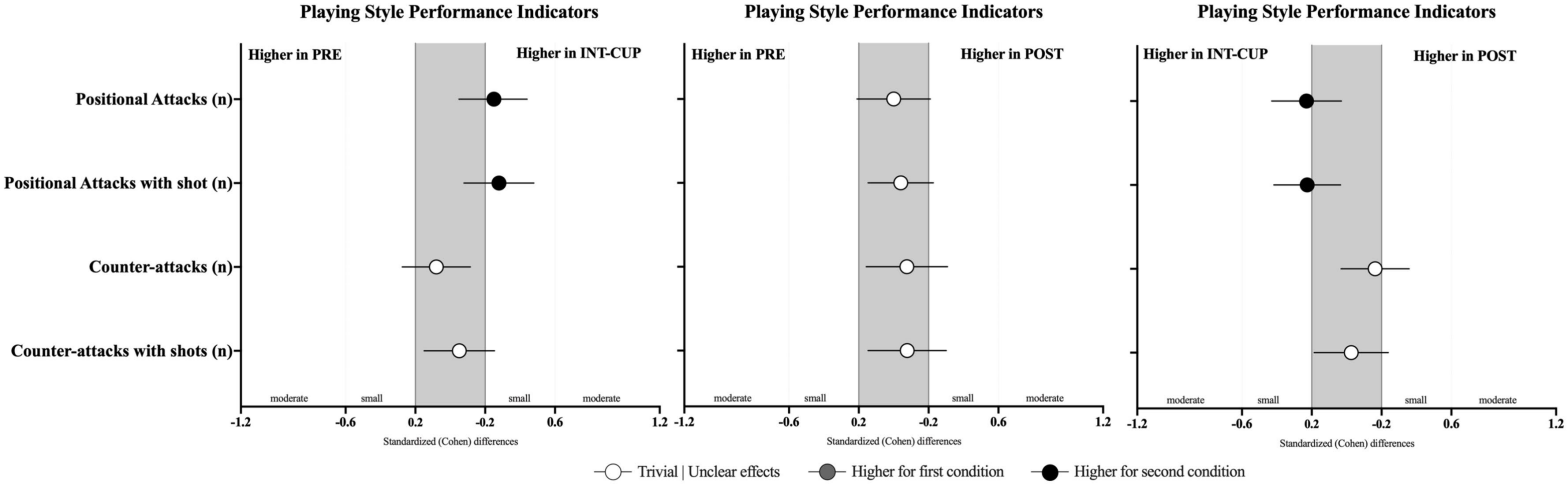
Standardized (Cohen’s *d*) differences in playing style variables across the conditions (PRE vs. INT-CUP, PRE vs. POST, and INT-CUP vs. POST). Error bars indicate uncertainty in the true mean changes with 95% confidence intervals.

**Figure 5:**
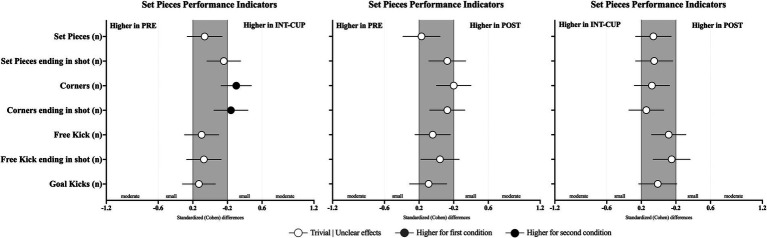
Standardized (Cohen’s *d*) differences in set pieces variables across the conditions (PRE vs. INT-CUP, PRE vs. POST, and INT-CUP vs. POST). Error bars indicate uncertainty in the true mean changes with 95% confidence intervals.

Differences in teams’ offensive playing style variables between the conditions (PRE vs. INT-CUP, PRE vs. POST, and INT-CUP vs. POST) are presented in Table 4 and Figure 4. Statistically significant differences were only observed for positional attacks ending with shots (*n*, *X*^2^ = 8.43, *p* = 0.015), with lower values in PRE compared to INT-CUP (*p* = 0.004; ES = 0.28 [0.08; 0.48]). No statistically significant differences were identified between the conditions for set pieces (see Figure 5).

**Table 4:** Descriptive and inferential statistics of offensive and set piece performance indicators across the conditions (PRE, INT-CUP, and POST).

Variables	PRE	INT-CUP	POST	Difference in means (raw; ±95% CL)			P	ES with 95% CI		
	(M ± SD)	(M ± SD)	(M ± SD)	PRE vs. INT-CUP	PRE vs. POST	INT-CUP vs. POST		PRE	INT-CUP	POST
Offensive playing style variables										
Positional attacks (n)	28.36 ± 9.18	30.84 ± 11.00	28.54 ± 9.57	2.48; ±1.93	0.01; ±2.06	−2.30; ±1.99	0.235	0.25 [0.05; 0.44]	0.00 [−0.21; 0.21]	−0.23 [−0.43; −0.03]
Positional attacks with shots (n)	7.10 ± 3.84	8.16 ± 4.19	7.30 ± 3.21	1.06; ±0.76	0.15; ±0.71	−0.86; ±0.73	**0.015**	0.28 [0.08; 0.48]	0.04 [−0.15; 0.23]	−0.23 [−0.42; −0.03]
Counter-attacks (n)	1.89 ± 1.93	1.74 ± 1.66	2.05 ± 1.96	−0.15; ±0.36	0.14; ±0.43	0.30; ±0.36	0.285	−0.08 [−0.28; 0.12]	0.08 [−0.16; 0.31]	0.16 [−0.03; 0.36]
Counter-attacks with shots (n)	0.79 ± 1.04	0.85 ± 1.16	0.88 ± 1.10	0.06; ±0.22	0.09; ±0.25	0.03; ±0.24	0.371	0.05 [−0.15; 0.25]	0.08 [−0.15; 0.30]	0.03 [−0.19; 0.24]
Set pieces variables										
Set pieces (n)	25.04 ± 4.73	24.7 ± 5.96	24.38 ± 5.23	−0.34; ±1.08	−0.92; ±1.15	−0.32; ±1.10	0.341	−0.06 [−0.26; 0.14]	−0.17 [−0.39; 0.04]	−0.06 [−0.27; 0.15]
Set pieces ending in shots (n)	3.52 ± 1.88	3.86 ± 2.19	3.75 ± 2.31	0.34; ±0.42	0.27; ±0.46	−0.11; ±0.46	0.561	0.16 [−0.04; 0.35]	0.13 [−0.09; 0.34]	−0.05 [−0.27; 0.16]
Corners (n)	4.62 ± 2.56	5.52 ± 3.07	5.29 ± 3.20	0.90; ±0.52	0.60; ±0.59	−0.23; ±0.61	0.096	0.30 [0.13; 0.48]	0.20 [0.00; 0.40]	−0.08 [−0.28; 0.13]
Corners ending in shots (n)	1.56 ± 1.35	1.91 ± 1.47	1.71 ± 1.52	0.35; ±0.29	0.18; ±0.30	−0.21; ±0.29	0.112	0.24 [0.04; 0.44]	0.13 [−0.08; 0.33]	−0.14 [−0.34; 0.06]
Free kicks (n)	2.26 ± 1.58	2.11 ± 1.54	2.29 ± 1.58	−0.16; ±0.31	−0.07; ±0.32	0.18; ±0.31	0.451	−0.10 [−0.3; 0.10]	−0.04 [−0.25; 0.16]	0.12 [−0.08; 0.32]
Free kicks ending in shots (n)	0.62 ± 0.88	0.56 ± 0.85	0.69 ± 0.89	−0.06; ±0.18	0.04; ±0.19	0.13; ±0.19	0.288	−0.07 [−0.27; 0.13]	0.04 [−0.18; 0.26]	0.15 [−0.06; 0.36]
Goal kicks (n)	7.59 ± 3.33	7.15 ± 3.42	7.11 ± 3.32	−0.44; ±0.65	−0.32; ±0.73	−0.04; ±0.73	0.225	−0.13 [−0.32; 0.06]	−0.09 [−0.31; 0.12]	−0.01 [−0.23; 0.21]

Bold values indicate statistically significant differences between the conditions: (a) statistically significant differences between PRE and INT-CUP; (b) statistically significant differences between PRE and Post; (c) statistically significant differences between INT-CUP and POST.

### Defensive performance indicators

**Figure 6:**
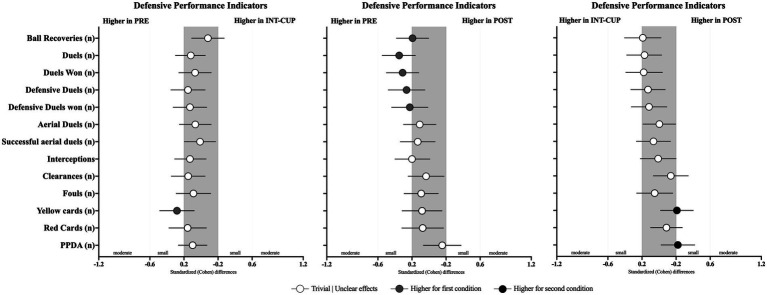
Standardized (Cohen’s *d*) differences in defensive performance variables across the conditions (PRE vs. INT-CUP, PRE vs. POST, and INT-CUP vs. POST). Error bars indicate uncertainty in the true mean changes with 95% confidence intervals.

Differences in teams’ defensive performance variables between the conditions (PRE vs. INT-CUP, PRE vs. POST, and INT-CUP vs. POST) are presented in Table 5 and Figure 6. Statistically significant differences between the conditions were observed only for duels (*n*, *X*^2^ = 10.8, *p* = 0.005), duels won (*n*, *X*^2^ = 8.47, *p* = 0.015), and defensive duels (*n*, *X*^2^ = 8.00, *p* = 0.018). In this respect, the PRE condition revealed higher values for duels and duels won compared to both INT-CUP (for duels, *p* = 0.037; ES = −0.12 [−0.3; 0.05]; and for duels won, *p* = 0.042; ES = −0.07 [−0.26; 0.12]) and POST (for duels, *p* = 0.001; ES = −0.35 [−0.55; −0.16]; and for duels won, *p* = 0.005; ES = −0.31 [−0.5; −0.12]). In addition, a higher number of defensive duels was observed in PRE compared to POST (*p* = 0.005; ES = −0.26 [−0.48; −0.05]).

**Table 5:** Descriptive and inferential statistics of defensive performance indicators across the conditions (PRE, INT-CUP, and POST).

Variables	PRE	INT-CUP	POST	Difference in means (raw; ±95% CL)			P	ES with 95% CI		
	(M ± SD)	(M ± SD)	(M ± SD)	PRE vs. INT-CUP	PRE vs. POST	INT-CUP vs. POST		PRE	INT-CUP	POST
Defensive performance variables										
Ball recoveries (n)	84.24 ± 12.77	85.40 ± 15.46	82.67 ± 14.33	1.16; ±2.74	−2.77; ±2.71	−2.73; ±3.07	0.591	0.08 [−0.11; 0.27]	−0.19 [−0.38; 0.00]	−0.19 [−0.41; 0.02]
Duels (n)	211.85 ± 28.47	207.8 ± 35.61	202.17 ± 33.65	−4.05; ±5.83	−11.68; ±6.29	−5.63; ±6.72	**0.005**	−0.12 [−0.30; 0.05]	−0.35 [−0.55; −0.16]	−0.17 [−0.38; 0.03]
Duels won (n)	101.82 ± 16.21	100.57 ± 18.86	97.45 ± 17.52	−1.28; ±3.33	−5.52; ±3.36	−3.12; ±3.76	**0.015**	−0.07 [−0.26; 0.12]	−0.31 [−0.50; −0.12]	−0.18 [−0.39; 0.04]
Defensive duels (n)	72.59 ± 14.98	70.22 ± 15.80	68.23 ± 15.30	−2.37; ±3.11	−4.07; ±3.32	−1.99; ±3.13	**0.018**	−0.15 [−0.35; 0.05]	−0.26 [−0.48; −0.05]	−0.13 [−0.33; 0.07]
Defensive duels won (n)	44.54 ± 10.51	43.21 ± 10.23	41.98 ± 10.45	−1.36; ±2.06	−2.36; ±2.23	−1.22; ±2.18	0.100	−0.13 [−0.33; 0.07]	−0.23 [−0.44; −0.01]	−0.12 [−0.33; 0.09]
Aerial duels (n)	34.31 ± 12.84	33.44 ± 13.32	33.48 ± 12.71	−0.87; ±2.43	−1.42; ±2.48	0.05; ±2.49	0.779	−0.07 [−0.25; 0.12]	−0.11 [−0.30; 0.08]	0.00 [−0.19; 0.19]
Successful aerial duels (n)	15.95 ± 6.96	15.87 ± 7.11	15.40 ± 7.19	−0.08; ±1.31	−0.94; ±1.46	−0.47; ±1.43	0.800	−0.01 [−0.19; 0.17]	−0.13 [−0.34; 0.07]	−0.07 [−0.27; 0.13]
Interceptions (n)	42.58 ± 9.58	41.26 ± 10.42	41.13 ± 10.68	−1.32; ±1.90	−2.03; ±2.10	−0.13; ±2.16	0.583	−0.13 [−0.31; 0.06]	−0.20 [−0.40; 0.01]	−0.01 [−0.22; 0.20]
Clearances (n)	16.01 ± 7.34	14.89 ± 7.96	15.92 ± 6.87	−1.12; ±1.48	−0.26; ±1.57	1.03; ±1.53	0.258	−0.15 [−0.35; 0.05]	−0.03 [−0.25; 0.18]	0.14 [−0.07; 0.34]
Fouls (n)	11.95 ± 3.87	11.60 ± 4.27	11.40 ± 3.61	−0.35; ±0.80	−0.36; ±0.79	−0.20; ±0.83	0.870	−0.09 [−0.29; 0.11]	−0.09 [−0.29; 0.11]	−0.05 [−0.26; 0.16]
Yellow cards (n)	2.21 ± 1.48	1.80 ± 1.41	2.10 ± 1.36	−0.40; ±0.29	−0.12; ±0.33	0.30; ±0.27	0.105	−0.28 [−0.49; −0.08]	−0.08 [−0.32; 0.15]	0.21 [0.02; 0.40]
Red cards (n)	0.13 ± 0.37	0.08 ± 0.29	0.11 ± 0.31	−0.05; ±0.07	−0.02; ±0.08	0.03; ±0.06	0.338	−0.16 [−0.38; 0.06]	−0.07 [−0.32; 0.17]	0.09 [−0.10; 0.27]
PPDA (n)	11.24 ± 5.09	10.71 ± 4.63	11.91 ± 6.32	−0.53; ±0.91	0.84; ±1.20	1.20; ±1.07	0.204	−0.10 [−0.27; 0.07]	0.15 [−0.07; 0.38]	0.22 [0.02; 0.42]

Bold values indicate statistically significant differences between the conditions: (a) statistically significant differences between PRE and INT-CUP; (b) statistically significant differences between PRE and Post; (c) statistically significant differences between INT-CUP and POST.

## Discussion

The aim of this study was to examine the impact of player absences resulting from participation in the AFCON and AC tournaments on team performance throughout the competitive season. Specifically, the investigation focused on how these absences affected metrics related to ball possession, goal scoring, offensive play, playing style, set pieces, and defensive actions.

According to tactical adaptation perspectives, teams tend to reorganize their structural and functional behaviors in response to temporary changes in player availability and contextual constraints (Lorenzo-Martínez et al., 2020). When key players are unavailable, teams tend to reorganize their tactical and structural behaviors to preserve stability and maintain performance. Evidence from other invasion sports supports this adaptive process. For example, research in professional hockey has shown that the loss of central players disrupts team interaction networks and requires functional reorganization to maintain effectiveness (Stuart, 2017). Similarly, studies in elite football have reported that teams increase their collective physical output, including sprints and high-speed running, when key players are absent (Windt et al., 2017). Together, these findings provide a theoretical basis to understand the tactical and physical adjustments observed in the present study.

Interestingly, teams’ performance during the INT-CUP period appears to be associated with improvements in ball possession and goal-scoring efficiency. In fact, a higher number of passes from different directions (i.e., lateral, frontal, backward) and types (i.e., deep, progressive, long) was observed during the INT-CUP period compared to the PRE and POST phases. In addition, there was an increase in total shots, shots on target, and goals. In contrast, most of the studied variables revealed similar values between the PRE and POST conditions.

### Effects of players’ absence (INT-CUP) on teams’ performance

Previous research has reported mixed effects of international duties on domestic performance, ranging from no significant difference in injury rates (Carling et al., 2015) to small negative impacts during AFCON participation (Perez, 2021). The overlap between international and domestic competitions presents challenges for clubs, potentially affecting season planning and game strategies. However, our findings challenge this assumption, revealing an increase in both the number (lateral, frontal, and backward) and types (deep and progressive) of passes during the INT-CUP phase compared to the PRE and POST phases.

Although the present study was not designed to test positional effects directly, the distribution of players by role (Table 1) indicates that a substantial proportion of absentees were midfielders and attacking players. These positions typically play a central role in ball circulation, progression, and involvement in transitional phases. Research indicates that substitutes, particularly midfielders and attackers, often demonstrate higher involvement in possession and passing actions compared to starters (Lorenzo-Martínez et al., 2021; Pan et al., 2023), which may help explain the increases observed during INT-CUP. Taken together, it is plausible that the substitute players in our sample, especially those operating in midfield and attacking roles, possessed technical profiles that may have contributed to the observed improvements in passing metrics and possession-based behaviors during the INT-CUP phase. Varmus et al. (2025) also noted that teams adjust their reliance on domestic and foreign players according to contextual demands, which supports the idea that available squad profiles influence passing behaviors during INT-CUP.

The observed increases in passing metrics, penalty area entries, and positional attacks during the INT-CUP phase align with the principles of controlled possession play. Possession-oriented play is linked to increased goal-scoring opportunities and improved passing efficiency (Yi et al., 2019b). Prolonged passing sequences (9 + passes) and progressive passes are also known to generate more shots and enhance scoring outcomes (Deb et al., 2024; O’Donoghue and Beckley, 2023). These mechanisms help explain the offensive improvements observed during the INT-CUP phase.

Defensively, the effects of player absences were less pronounced. Defensive metrics remained largely stable, suggesting that defensive organization depends more on collective coordination than on individual contributions (Welch et al., 2021). The possession-oriented adjustments may also have reduced defensive workload, consistent with findings that possession-heavy teams defend less (Yamada and Hayashi, 2015).

In general, while player absences due to the AFCON and AC tournaments may initially be perceived as detrimental, our findings indicate a shift in playing style that appears to enhance offensive metrics, particularly passing performance. This suggests that teams can adapt strategically by incorporating alternative players with complementary skill sets and adjusting tactical structures. In this respect, Varmus et al. (2025) emphasized that teams strategically manage the balance between domestic and foreign players to maintain squad depth and adaptability. Our results reinforce this notion by suggesting that the absence of key players during international tournaments may prompt coaches to reassess their tactical structures, often leading to an increase in possession-based play. Conversely, defensive stability appears to be less affected, reinforcing the idea that defensive organization is more system-oriented than individually dependent. However, the lack of studies specifically analyzing the impact of player absences during these international tournaments limits the ability to directly compare our findings with prior research. Most existing literature has focused on broader impacts, such as team performance outcomes or economic consequences, rather than in-game technical and tactical adaptations. For instance, Perez (2021) examined the effects of player absences during the AFCON from a performance standpoint, concluding that team success was negatively affected. However, his study did not account for technical performance indicators, such as passing dynamics and offensive structures, which our research highlights as key adaptive mechanisms. Therefore, this study adds novel insights to the existing body of literature by demonstrating that, beyond overall team performance, strategic and tactical adjustments may help mitigate the loss of key players and could even be associated with improvements in specific offensive metrics. Future research should further explore the nuanced effects of player absences in different contexts, considering not only performance outcomes but also tactical and technical responses, to better inform coaching strategies and squad management during overlapping international competitions.

### Effects of players’ return (PRE vs. POST comparison) in teams performance

Losing players to international duties is often associated with disruptions in team performance, particularly in competitive leagues where squad depth plays a crucial role (Perez, 2021). Although our findings revealed distinct shifts in team dynamics during the AFCON and AC tournaments, particularly in passing efficiency and goal-scoring metrics, the differences between the PRE and POST phases were less pronounced. Specifically, the PRE phase showed greater use of long successful passes, longer average passing distances, and more successful crosses compared to the POST phase, suggesting a shift away from direct play after the tournament. As highlighted in previous research, player absences may have necessitated tactical adaptations, often resulting in a more controlled, possession-oriented style (Memmert et al., 2019; Yi et al., 2019b). In this case, the tactical adjustments observed during the tournament, such as increased short passing and offensive volume, likely contributed to higher goal-scoring efficiency (Deb et al., 2024; O’Donoghue and Beckley, 2023). Given the effectiveness of this adjusted style, it is plausible that even after the return of international players, coaches opted to maintain a more possession-based approach, which led to a reduction in the frequency of long passes and crosses in the POST phase. This aligns with previous findings suggesting that teams strategically adjust their playing style not only in response to player absences but also based on observed in-game efficiencies (Forcher et al., 2022).

From a defensive perspective, the higher number of duels, duels won, and defensive duels observed in the PRE phase may have been a direct consequence of the earlier adoption of long passes. Long-ball strategies typically lead to more frequent aerial duels, second-ball battles, and transitional defensive actions, as the ball is contested more often in open spaces rather than retained through controlled build-up play. This strategy typically results in more frequent losses of ball possession, with one study finding that 59% of long passes led to possession loss, while only 1% resulted in shots on goal (dos Reis et al., 2017). The effectiveness of long passes may have been further diminished by the evolution of soccer toward higher player density and increased passing rates (Wallace and Norton, 2014). Therefore, the observed decrease in defensive duels in the POST phase may reflect an effort to maintain the possession-based style introduced during the INT-CUP phase.

Overall, this suggests that while teams undergo tactical adjustments and performance fluctuations during tournaments, their playing style and effectiveness tend to stabilize once the full squad is reinstated. The relatively minor changes observed between PRE and POST indicate that any tactical adaptations or performance shifts induced by player absences are likely temporary rather than representing long-term transformations.

Although this study provides valuable insights into the impact of player absences during international tournaments on team performance, several limitations should be acknowledged. The analysis was restricted to a single season and a specific set of teams affected by the AFCON and AC tournaments, which may limit the generalizability of the findings to other leagues or competitions with distinct tactical demands and playing styles. Individual-level factors, such as player experience, physical attributes, and tactical roles, were not included in the analysis, which may have influenced the observed adaptations. Contextual variables, including match importance, opposition quality, and in-game tactical adjustments, were not controlled for, yet they may have significantly affected team performance across the PRE, INT-CUP, and POST phases. In addition, coach-level data and team formation details were not considered, which may have confounded the interpretation of tactical and strategic adjustments. Broader squad-related factors, such as team ability, depth, injury status, and overall characteristics, were likewise not included and may represent additional sources of variation. Future research should include a broader range of teams, consider individual and contextual variables, and analyze these adaptive processes across multiple seasons to better understand how teams respond to international tournament absences.

## Conclusion

This study highlights that teams adapted to player absences during international tournaments by adopting a more possession-based style of play, leading to increased passing volume, goal-scoring efficiency, and offensive play. These tactical adjustments contributed to a decrease in the number of long passes and crosses and, consequently, fewer defensive duels when comparing the PRE and POST phases. The findings suggest that player absences trigger short-term tactical adjustments rather than long-term structural transformations. From a practical standpoint, coaches and performance analysts should view forced squad rotations as opportunities to explore alternative tactical frameworks that may enhance offensive efficiency while maintaining defensive stability.

### Variation in passing behavior before, during, and after the two competitions

Our findings reported that passing-related variables showed the most statistically significant differences, particularly during the INT-CUP period, which exhibited a higher number of passes across nearly all variables analyzed. An exception was observed for long passes and successful long passes, which did not show a positive effect during the INT-CUP period compared to the PRE period. This may be attributed to substitute players’ tendency to avoid risk, as long passes are inherently associated with a higher probability of error. Supporting this interpretation, dos Reis et al. (2017) showed a proportional relationship between the frequency of long-distance passes and ball possession loss; that is, the longer the passes attempted, the higher the likelihood of losing the ball. Notably, upon the player’s return (POST period), long passes and successful long passes were higher during the INT-CUP period compared to the POST period. In addition, the average passing distance was greater in the PRE phase compared to the INT-CUP period.

An increase in the number of crosses and successful crosses was observed when comparing the POST and PRE periods with the PRE period. According to Yamada and Hayashi (2015), the compact defensive blocks used in modern soccer make wide attacks particularly effective, as they enable teams to deliver crosses into high-probability scoring zones, such as the prime target area. This increase may be attributed to the number of players occupying wide positions on the field, compared to other positions, who were called up for national teams, as shown in Table 1. In addition, this may be due to substitute players being afraid of losing the ball or making mistakes. In high-competition environments with larger audiences, athletes’ perceptions of their mistakes and performances as failures can have negative consequences, especially for those who are concerned about errors and others’ negative evaluations (Sagar et al., 2010). Yamada and Hayashi (2015) reported that attacks developed through the wings frequently lead to goal-scoring situations, with crosses into dangerous central areas being particularly effective. This may also explain why more offensive duels were observed during the PRE period compared to the POST period.

### Offensive performance indicators before, during, and after the two competitions

Previous studies have shown that goals are the decisive factor in determining match outcomes and distinguishing top-performing teams from the rest (Griszbacher, 2024). Our findings revealed that offensive performance variables, including total shots, shots on target, shots on target from outside the penalty area, goals following penalty area entries, penalty area touches, corners, and corners ending in shots, exhibited the most statistically significant differences during the INT-CUP period. This may be attributed to the fact that offensive passing variables, such as deep completed passes and final third successful passes, were also consistently high during the INT-CUP period. Gonzalez-Rodenas et al. (2020) highlighted the importance of penetrative passes for creating goal-scoring opportunities. In addition, while short penetrative passes lead to more scoring opportunities, long penetrative passes are particularly effective in disrupting defensive organization (Zani et al., 2021).

### Defensive performance indicators before, during, and after the two competitions

According to our results, defensive performance indicators were higher in the PRE period compared to the POST period for variables such as duels, duels won, defensive duels, and defensive duels won. This may be attributed to the greater availability of information about the opposition, such as video analysis, which allows coaches and players to better understand the opponents’ style of play. By watching past matches of the opposing team, players can identify strengths, weaknesses, game tactics, and patterns, allowing them to better prepare for the match (Iulian et al., 2024). As a result, players can anticipate their opponents’ actions and be more aware of their strategies, increasing their chances of winning duels.

Despite these results, several limitations must be acknowledged. First, data collection was limited to a single season of the AFCON and AC competitions. Although the sample size was substantial, it was restricted to just one season. Second, the statistical tool used for data collection underwent rebranding during the study. Finally, there is a lack of prior research on this topic, highlighting the relevance and need for further studies.

This study can assist coaching staff in preparing for matches during periods when players are called up for national team duties in the AFCON and AC competitions. It also provides valuable insights into player behavior in the absence of teammates participating in these tournaments, allowing training adjustments to address the specific demands and characteristics of matches during these periods.

## Data Availability

The datasets presented in this article are not readily available to protect the subjects’ confidentiality and privacy. Requests to access the datasets should be directed to the board from the Research Center in Sports Sciences, Health Sciences, and Human Development (cidesd.geral@utad.pt).
